# Experience of Pain and Unpleasantness during Mammography Screening: A Cross-Sectional Study on the Roles of Emotional, Cognitive, and Personality Factors

**DOI:** 10.3390/bs13050377

**Published:** 2023-05-04

**Authors:** Casandra I. Montoro, María del Carmen Alcaraz, Carmen M. Galvez-Sánchez

**Affiliations:** 1 Department of Psychology, University of Jaén, 23071 Jaén, Spain; 2 Diagnostic Mammography Unit, Health Center of Martos, Distrito Jaén Sur, 23600 Jaén, Spain

**Keywords:** pain, unpleasantness, mammography screening, psychoticism, state anxiety

## Abstract

Background: Breast cancer is the most frequent cause of malignant tumors among women worldwide. Its successful prevention depends on the degree of participation in screening programs, which can be influenced by psychological factors, including fear. Method: A cross-sectional study was conducted according to the Strengthening the Reporting of Observational Studies in Epidemiology (STROBE) Statement. Twenty-six healthy women aged 50–69 years took part in this study, all of whom were summoned for routine mammography screening and were randomly selected. Prior mammography screening, breast pain intensity, unpleasantness (visual analog scale), and psychological (catastrophizing, state anxiety, and fear of pain) and personality (neuroticism, psychoticism, and extraversion) variables were evaluated. Pain, unpleasantness, and state anxiety were further evaluated pre- and post-mammography screening. Results: During the mammography screening, pain and unpleasantness levels were higher than those observed pre- and post-screening. Residual unpleasantness remained post-screening. State anxiety was positively associated with pain, and psychoticism with unpleasantness, as reported by participants during the mammography screening. Conclusions: Anxiety levels influence the pain experienced in association with the mammography procedure. Women subjected to mammography screenings might benefit from relaxation strategies aimed at reducing anxiety to pre-mammography levels and, by extension, pain and unpleasantness during mammography. The inclusion of these strategies in breast cancer prevention campaigns could improve the rates of mammography reattendance, and therefore, benefit cancer prevention efforts.

## 1. Introduction

Breast cancer is an important challenge for healthcare systems because it is both the most frequent cause of malignant tumors among women worldwide [1] and the most common cancer in developed and developing regions [2]. Moreover, breast cancer is the leading cause of mortality among women worldwide [3]. In 2020, >2 million new breast cancer cases were diagnosed (23% of all cancers), and around 685,000 women died as a consequence of this disease [3]. According to the World Health Organization [3], 1 in 12 women will experience breast cancer during their lifetime.

According to general epidemiological statistics, breast cancer incidence and mortality vary according to a country’s degree of development. In developed countries and regions (i.e., Australia/New Zealand, North America, and parts of Europe), the incidence of breast cancer tends to be higher compared to that in developing countries (i.e., Asian and African countries) [4,5]. Relative mortality, although less geographically variable, is also greater in less developed countries [4,5].

Regarding classification, the Union for International Cancer Control (UICC; [6]) classifies breast cancer into four stages according to its extensiveness: (a) Stage I: small tumors without metastatic involvement of the axilla, (b) Stage II: tumors > 2 cm or moderate metastatic involvement of the axilla, (c) Stage III: very large tumors, skin or pectoral muscle involvement, or massive axillary involvement, and (d) Stage IV: metastasis in distant organs (bone, lung, liver, etc.).

The prognosis differs markedly among stages; the approximate 5-year survival rates are 95% (stage I), 80% (stage II), 60% (stage III), and 25% (stage IV) [2,6]. Advanced-stage disease necessitates a more challenging, uncertain, and aggressive treatment, which is typically pharmacological [7]. The diagnosis of breast cancer in its early stages is crucial to reducing physical and psychological sequelae and increasing the likelihood of a cure [8,9,10].

Breast screening programs are intended to detect the disease in its preclinical phase, and thus, reduce morbidity and mortality rates [11,12,13]. Mammography, which is widely available in many countries (e.g., the United Kingdom, the United States, Germany, Italy, and Spain [13,14,15]), is the technique of choice for the early diagnosis of breast cancer [13]. Biennial screening in asymptomatic 50–69-year-old women is considered the most favorable approach [16]. Unfortunately, in countries with limited resources (e.g., India, Mexico, Brazil, Gambia, etc.) breast screening programs are lacking or are constrained by economic factors (e.g., private health care accessibility). Therefore, breast cancer is commonly diagnosed at late stages, which accounts for the greater mortality seen in developing countries [17,18].

Mammography studies include two projections: craniocaudal (CC) and medial lateral oblique (MLO). Both projections are made by pulling and separating the breast tissue from the chest wall [19]. Mammographers incorporate a blade to compress the breast, with the aim of immobilizing the tissues and reducing breast thickness. This system reduces the radiation dose and improves image quality [20]. However, 75% of women describe mammography screening as a painful experience, and as a factor influencing the perceived quality of the service and the success of early detection programs [21]. Some women even report feeling discomfort and/or pain in subsequent days [22]. In this sense, cumulative participation is the most important factor in determining the success of screening programs and, by extension, breast cancer prevention campaigns [23]. Pain experience is considered an important factor influencing subsequent attendance to mammograms [21,22,23,24]. According to a systematic review conducted by Whelehan et al. (2013) [23], women who have not previously experienced pain are more likely to re-attend than those who have.

Pain is a subjective experience influenced by biological, psychological, and socio-cultural factors [25]. Among the psychological features, catastrophizing has been proven to be linked to pain experience in healthy and clinical populations [26]. In the context of breast cancer, catastrophizing has mostly been investigated in relation to adherence [27], the experience of persistent breast and mammography pain in breast cancer survivors [28,29], and perceived risk and concerns about recurrence among breast cancer survivors’ relatives [30]. Although the literature on catastrophizing and mammography-related pain is relatively scarce, some authors, such as Asghari & Nicholas (2003) [31], demonstrated that greater use of catastrophizing and coping self-statements predicted more severe pain during mammography. However, the data were collected from women undergoing screening mammography in a private clinic, where unequal access to public and private healthcare services due to income disparities has been proposed as a predictor of compliance with mammographic screening [32].

State anxiety and fear of pain are additional psychological factors that are widely associated with subjective pain experience [33,34]. Anxiety has been considered the most common psychologically distressing consequence of mammography, whereas fear is considered to act as a subjective psychological barrier to undergoing mammography [35].

Similarly, personality traits (through several mediating mechanisms, i.e., coping strategies, lifestyle, reactivity, etc.) are also known to modulate psychosocial factors affecting pain experience [36]. Eysenck’s personality theory is one of the most empirically well-supported personality theories. It comprises three personality dimensions: extraversion, neuroticism, and psychoticism [37]. Extraversion—a personality trait defined by dynamism, positive emotionality, interest in the outside world, and sociability—has been proposed as a protective factor against pain [38,39]. The protective role of extraversion against pain may be explained by its influence on selective attention toward pain [40].

Neuroticism—aA trait characterized by a stable tendency to experience negative emotions—is associated with a greater number of catastrophic thoughts about pain in chronic pain patients [41,42], as well as higher overall levels of pain both in sufferers of chronic pain and healthy individuals [38]. Psychoticism—or the propensity for tough-mindedness, impulsivity, aggressiveness, egocentrism, low empathy, anti-sociability, and creativity—has been associated with greater use of catastrophism as a pain coping strategy in healthy individuals [38,43]. Concerning cancer, studies have largely focused on the association between these personality traits and screening compliance [44].

Despite the relevance of mammography-related pain to the effectiveness of prevention campaigns and the influence of biopsychosocial factors on that pain, few studies have explored the aforementioned factors in relation to mammography-related pain in healthy women undergoing screening mammography. Likewise, studies conducted in public health settings are quite scarce. Given the above, the present study aimed to: (1) analyze changes in pain ratings associated with routine mammography screening in a public health center and, (2) explore the influence of psychological variables (personality, state anxiety, catastrophizing, and fear of pain) on pain and unpleasantness during mammography screening.

It is hypothesized that women will experience high levels of pain and unpleasantness during screening, and that psychological variables such as state anxiety, catastrophizing, and personality modulate these perceptions. Further insights into mammography-related pain and unpleasantness may be relevant both clinically and for prevention efforts. This research intends to improve our understanding of the mammography experience, with the purpose of reducing the associated discomfort and increasing adherence to biennial screening.

## 2. Materials and Methods

### 2.1. Participants

Thirty women were asked to participate in the study; only four declined to participate (because the study clashed with their work schedules). In total, 26 healthy women aged 50–69 years took part in this study. All of the participants were summoned for a routine mammography screening at the Centro de Salud Bulevar (Jaén, Andalusia), and were randomly selected from the Breast Cancer Detection (DCM) appointment lists for Jaén’s health district. The exclusion criteria for all participants were metabolic abnormalities, neurological disorders (e.g., traumatic head injury), and other severe somatic (e.g., cancer, including breast cancer) or psychiatric (e.g., drug dependency or psychosis) diseases. No women had clinical signs of breast pathology or breast implants. The participants’ sociodemographic data are shown in Table 1. According to the Strengthening the Reporting of Observational Studies in Epidemiology (STROBE) Statement [45] (see Section 2.2), previous variables used to characterize the sample (i.e., the number of cohabitants and children, which has important implications for mammography attendance [46]) and potential confounding factors (i.e., menopause and breast size) are also displayed in Table 1. All participants voluntarily consented to participate in the study.

### 2.2. Procedure

This cross-sectional study was conducted based on the STROBE Statement: guidelines for reporting observational studies [45]. Specifically, the STROBE checklist: cross-sectional studies guidelines was used to enhance the quality and transparency of the research (see Supplementary Materials for further details; Table S1). To reduce possible bias, after randomizing the sample, participants were assigned a code to ensure blinding of both the radio diagnostic technician (M.C.A.), who collected the data, and the authors, who conducted the statistical analyses. In the first phase—prior to the mammography screening—breast pain intensity and unpleasantness were evaluated using a visual analog scale (VAS), along with psychological (catastrophizing, state anxiety, and fear of pain) and personality (neuroticism, psychoticism, and extraversion) variables, via self-report questionnaires. In the second phase, i.e., during the screening, the intensity and unpleasantness of the pain in each mammographic projection (CC and MLO) was measured using the VAS scale. Immediately after the study of the breasts was completed, the level of state anxiety and possible residual breast pain (intensity and unpleasantness) were reevaluated.

The protocol was approved by the provincial research ethics committee of Jaén (Junta de Andalucía; Consejería de Salud y Provincias (TFG-DMS-2021/1228-N-21)).

### 2.3. Psychological Measures

In addition to an interview used to obtain demographic data, the participants were evaluated using the below self-report questionnaires:

Spanish Adaptation of the Eysenck Personality Questionnaire Revised—Abbreviated (EQR-A; Sandín et al., 2002) [47]. This truncated questionnaire consists of 24 items (6 items per scale) and explores the three personality dimensions: extraversion (E), neuroticism (N), and psychoticism (P); it also includes a sincerity scale (L). Scores range between 0 and 6 on all subscales (answer format = Yes/No). The Cronbach’s alpha was 0.83 for extraversion, 0.77 for neuroticism, 0.62 for psychoticism, and 0.81 for sincerity in women [48].

Spanish Adaptation of the State–Trait Anxiety Inventory (STAI; Spielberger et al., 1982) [49]. This instrument consists of 40 items measuring state (E) and trait (R) anxiety. Scores range between 0 and 60 for both state and trait anxiety. In the present study, we only evaluated state anxiety, for which Cronbach’s alpha ranged between 0.90 and 0.93 [49].

Fear of Pain Questionnaire III (FP-III; McNeil & Rainwater, 1998) [50]. This instrument consists of 30 items and three subscales (severe pain, mild pain, and medical pain) assessing fear (alarm reaction) of pain in different situations on a 5-point Likert scale ranging from 1 (Not at all) to 5 (Extreme). The Cronbach’s alpha was 0.70 [51].

Spanish adaptation of the Coping Strategies Questionnaire (CSQ; Rodriguez et al., 2004) [52]. This 39-item questionnaire evaluates the frequency of use of both adaptive and maladaptive pain coping strategies. In this study, only the six-item “catastrophizing” pain subscale was used (score range: 0–36). The Cronbach’s alpha was 0.89 for catastrophizing [52].

### 2.4. Data Analysis

In the first step, the independent effects of mammography screening on pain and unpleasantness were analyzed using 3 (time: pre-, during, and post-mammography screening) × 2 (side: left and right) repeated-measures ANOVAs. The mean value of the projections (i.e., CC and MLO) was computed for breast pain and unpleasantness during mammography screening for the above analyses. In the second step, *t*-tests were used to compare state anxiety before and after mammography screening. In the third step, the independent effects of mammography screening projections on pain and unpleasantness were analyzed using 3 (projections: CC and MLO) × 2 (side: left and right) repeated-measures ANOVAs. Finally, Pearson bivariate correlations of personality (neuroticism, extraversion, and psychoticism), psychological variables (catastrophizing and fear of pain), and pain and unpleasantness during mammography screening (using the mean value of the projections) were performed. The level of significance was set at *p* ≤ 0.05 (two-tailed). The Huynh–Feldt epsilon correction was applied for adjustment of the degrees of freedom, and post hoc Bonferroni correction was used if necessary. There were no missing data.

## 3. Results

### 3.1. Effects of Mammography Screening on Breast Pain, Unpleasantness, and State Anxiety

Regarding breast pain, significant main effects of time and side were observed (Table 2). Post hoc comparisons revealed that pain during mammography was more severe compared with that both pre- and post-screening. Marginal differences were observed between pre- and post-screening pain (Table 3). Pain in the left breast was more severe than in the right (1.58 ± 0.166 vs. 1.38 ± 0.156; *p* = 0.016).

For unpleasantness, the only significant main effect was that of time (Table 2). Post hoc comparisons revealed that unpleasantness during mammography was higher compared with that both pre- and post-screening. After mammography, unpleasantness remained higher than that experienced pre-screening (see Table 3).

State anxiety was higher pre- (17.07 ± 10.44) than post- (13.23 ± 8.86) mammography screening (t = 2.64; *p* = 0.014).

### 3.2. Effect of Mammography Screening Projections on Breast Pain and Unpleasantness

Upon comparing mammography screening projections (i.e., CC and MLO), no significant main effect of projection or side was seen, and there was no significant projection × side interaction effect for either pain or unpleasantness (see Table 4).

### 3.3. Associations of Psychological Variables, Pain, and Perceived Unpleasantness during Mammography Screening

Among the psychological variables explored in this study, only state anxiety and psychoticism were correlated with pain and unpleasantness during mammography screening. State anxiety was positively associated with pain, whereas psychoticism was positively associated with both pain and unpleasantness. No significant associations were observed for fear of pain or catastrophizing (see Table 5).

## 4. Discussion

The present study aimed to analyze changes in pain and unpleasantness ratings associated with the mammography procedure, and to explore the influence of psychological variables—personality, state anxiety, catastrophizing, and fear of pain—on pain and perceived unpleasantness during routine mammography screening in a public health center.

Congruent with previous findings [21,22,23,24,53], pain and unpleasantness ratings during mammography were higher compared with those both pre- and post-screening. Although pain post-mammography returned to its initial level, unpleasantness post-mammography remained higher, confirming a residual feeling of discomfort or displeasure even after screening is accomplished [22].

Regardless of the time point (pre, during, or post-screening), pain in the left breast was greater than in the right. Additionally, pain did not differ between the left and right sides when projections (CC and MLO) were analyzed. In light of these results, differences in pain between breasts were explained neither by the mammography procedure nor the type of projection. A plausible hypothesis is greater intrinsic sensitivity of the left breast compared to the right. However, no previous studies of healthy women are available to support this assumption. Studies have mostly explored breast sensitivity in distinct cutaneous areas [54], or after interventions such as mastectomy, implant-based breast reconstruction [55,56], and reduction mammaplasty [57,58,59]. Uncontrolled factors in our study could also account for the greater left breast pain, such as breast size [60,61], or more specifically, left breast size.

With respect to psychological factors, state anxiety was higher pre- than post-mammography screening. The prompt decline in anxiety after screening indicates that the women felt more relaxed. This is congruent with state anxiety as a future-oriented emotional state characterized by anticipatory affective, behavioral, and cognitive reactions to potential threat and uncertainty [62]. Notwithstanding the foregoing, and as reported above, state anxiety prior to screening was positively associated with mean pain ratings during mammography screening. Therefore, congruent with previous studies on pain perception [33,34], anxiety was likely to have an effect on posterior pain perception during the screening. Opposite to Loving et al. (2021) [35], anxiety was not considered a psychologically distressing consequence of mammography, but rather, a psychological factor prior to mammography that influenced the pain experience. Congruently, Fazeli et al. (2021) [53] observed that a considerable percentage of women experienced fear and anxiety before (58%) and during (49%) a magnetic resonance imaging breast examination.

In this study, psychoticism was related to mean unpleasantness during mammography screening. Among the personality traits evaluated, psychoticism was the one for which we had the fewest expectations. Indeed, in one of our previous studies [38], neuroticism was the only personality trait positively associated with pain and state anxiety in healthy individuals; psychoticism did not show any relation to pain. The lack of congruency in the findings of the present study and our previous study may relate to the type of pain and method used for its evaluation (mammography-related breast pain according to VAS scores vs. non-localized pain assessed via questionnaire). Nonetheless, unpleasantness is distinct from sensory pain. It is an emotional experience (i.e., a disturbing emotion) resulting from pain and mediated by memories and contextual features. In a study conducted by Rawlings (2003) [63], a preference for unpleasant paintings in students with high psychoticism scores was observed. Although no firm conclusions can be drawn from these results, it can be hypothesized that individuals scoring higher in psychoticism might tend to seek and experience unpleasantness. In the present study, no correlation was observed between psychoticism and pain catastrophizing (r = −0.193, *p* = 0.345), which, together with the lack of associations of catastrophizing with the variables that we evaluated, would rule out any role of catastrophizing in explaining our results, in contrast to the study of Asghari and Nicholas (2003) [31].

Other factors (e.g., self-efficacy, susceptibility, motivation, and perceived barriers) have been associated with greater or lower rates of screening attendance [64], and could be related to mammography pain experience. Certain factors have been shown to be associated with the capacity to tolerate discomfort during mammography, such as breast mass, abnormal test results, and the consumption of substances containing methylxanthine [65]. Given that avoidance of discomfort has been associated with increased pain perception [65], future studies addressing these factors in relation to pain experience are highly encouraged.

The present results show the complexity of the pain experience and psychological factors related to mammography screenings. Given the (1) effect of anxiety on pain perception, (2) well-reported association between pain perception and cumulative participation in mammography screening, and (3) relevance of this cumulative participation to the prevention of breast cancer, effective pain-reducing interventions for mammography are needed to reduce the associated anxiety. Women subjected to mammography screenings may benefit from relaxation strategies (e.g., breathing and visual thinking exercises) aimed at reducing anxiety to pre-mammography levels. Psychoeducation programs aimed not only at patients and women at risk, but also at healthy women, could also be useful for improving adherence to mammography screening [66,67]. A major challenge to social health systems is making women more aware of the benefits of early examinations, with the goal of encouraging them to include mammography in their health routines [68]. When women feel more confident and less stressed, the reattendance rate is likely to increase.

In relation to the above, the rate of breast cancer survival for at least 5 years after diagnosis varies from over 90% in high-income countries to 66% and 40% in India and South Africa, respectively [3]. In high-income countries, early detection and treatment have achieved good results. Consequently, the World Health Organization (WHO) launched the Worldwide Initiative against Breast Cancer, whose main objective is to reduce global breast cancer-related mortality by 2.5% per year. If this goal is met, 2.5 million fewer deaths worldwide are expected from 2020 to 2040. The three pillars needed to accomplish this objective are: (1) health promotion for early detection, (2) timely diagnosis, and (3) comprehensive management of breast cancer [3]. The present findings are in line with this initiative; they highlight the importance of designing and promoting interventions aimed at reducing the pain and unpleasantness associated with mammography to accomplish the WHO’s objective.

The main limitations of the present study were Its cross-sectional design, which did not allow for the establishment of causal associations, as well as the small sample size and failure to correct for Type I errors. Furthermore, the effects of breast size and/or menstrual cycle on breast pain and unpleasantness were not accounted for. Breast size and breast asymmetry have been suggested as risk factors for breast cancer [69], and it has also been stated that the first factor has a significant impact on breast pain experience [61,62]. Additionally, cyclical mastalgia (i.e., cyclical breast pain) has been associated with the menstrual period [70]. Although no data on the menstrual cycle were collected in the present study, breast size was measured. However, the small sample size did not allow for subgroup comparisons. Lastly, the anti-sociability—and consequently, the likelihood of fewer social contacts—that characterizes the psychoticism personality trait might negatively influence the mammography experience and reduce the benefits of mammography screening programs. In this regard, social support (as indicated by cohabitation, for example) has been associated with a greater probability of attending and reattending mammography exams [46,71,72,73]. To draw firm conclusions, further studies assessing the effects of these variables on breast pain during mammography screening, and addressing the other limitations mentioned above, are needed.

## 5. Conclusions

In summary, the levels of pain and perceived unpleasantness during mammography were higher compared to those both pre- and post-screening. State anxiety was higher pre-mammography screening compared to post-screening. Residual unpleasantness was observed post-screening. During screening, state anxiety was positively associated with pain, and psychoticism with unpleasantness. These findings highlight the relevance of psychological factors to mammography exams, and confirm the need for programs aiming to enhance breast cancer prevention, diagnosis, and treatment to consider these factors. A better understanding of the experience of mammography screenings and associated psychological factors might facilitate the development of personalized biopsychosocial treatment plans to optimize the early detection and prevention of breast cancer [74,75].

## Figures and Tables

**Table 1:** Participants’ sociodemographic characteristics and confounding factors.

	Categories	M ± SD or n (%)
Age	-	58 ± 5.59
Years of education	-	11.53 ± 4.67
Menopause	Yes	4 (15.4)
	No	22 (84.6)
Breast size *	Small	15 (57.7)
	Medium	8 (30.8)
	Large	3 (11.5)
Number of cohabitants	-	2.81 ± 0.85
Number of children	-	2.00 ± 1.23

Note. Years of education = number of years of education according to the Spanish educational system. * Breast size was established according to the surface area of compression blades occupied by the breasts.

**Table 2:** Repeated-measures ANOVAs for the effects of mammography screening on pain and unpleasantness.

	Effects	F (df) *	*p*	ŋp²
Breast pain	Time	85.56 (1.02, 25.72)	**<0.0001**	0.774
	Side	6.69 (1.00, 25.00)	**0.016**	0.211
	Time × side	1.55 (1.44, 36.00)	2.226	0.059
Unpleasantness	Time	40.14 (1.03, 25.77)	**<0.0001**	0.616
	Side	2.06 (1.00, 25.00)	0.164	0.076
	Time × side	1.20 (1.52, 38.16)	0.302	0.046

Note. * Degrees of freedom (df) were adjusted via Huynh–Feldt epsilon correction. Significant *p* Values are highlighted in bold.

**Table 3:** *p*-values for post hoc comparisons of the main effect of time on pain and unpleasantness.

			*p* Value		
	Time	M ± SD	Pre	During	Post
Pain	Pre	0.019 ± 0.019	-	**<0.0001**	0.096
	During	4.279 ± 0.454	**<0.0001**	-	**<0.0001**
	Post	0.154 ± 0.061	0.096	**<0.0001**	-
Unpleasantness	Pre	0.000 ± 0.000	-	**<0.0001**	**0.028**
	During	3.173 ± 0.494	**<0.0001**	-	**<0.0001**
	Post	0.192 ± 0.068	**0.028**	**<0.0001**	-

Note. Pre = pre-mammography screening; During = during mammography screening; Post = post-mammography screening. Significant *p* Values are highlighted in bold.

**Table 4:** Repeated-measures ANOVAs for the effects of mammography screening projections (craniocaudal and mediolateral oblique) on breast pain and unpleasantness.

	Effects	F (df) *	*p*	ŋp²
Breast pain	Projection	0.34 (1.00, 25.00)	0.563	0.014
	Side	3.16 (1.00, 25.00)	0.088	0.112
	Projection × side	3.73 (1.00, 25.00)	0.065	0.130
Unpleasantness	Projection	0.52 (1.00, 25.00)	0.480	0.020
	Side	1.50 (1.00, 25.00)	0.232	0.057
	Projection × side	1.65 (1.00, 25.00)	0.210	0.062

Note. * Degrees of freedom (df) were adjusted via Huynh–Feldt epsilon correction.

**Table 5:** Pearson bivariate correlations of psychological variables, pain, and unpleasantness during mammography screening.

Psychological Measures		Pain	Unpleasantness
EPQR-A	Neuroticism	0.058	−0.120
	Extraversion	0.332	0.310
	Psychoticism	0.272	0.467 *
STAI	State Anxiety	0.389 *	0.205
FP-III	Fear of severe pain	0.219	0.248
	Fear of mild pain	0.107	0.134
	Fear of medical pain	0.213	0.138
CSQ	Catastrophizing	0.109	−0.035

Note. * *p* < 0.05; EPQR-A = Spanish Adaptation of Eysenck Personality Questionnaire Revised—Abbreviated; STAI = Spanish Adaptation of State–Trait Anxiety Inventory; FP-II = Fear of Pain Questionnaire-III; CSQ = Spanish adaptation of Coping Strategies Questionnaire. Significant p Values are highlighted in bold.

## Data Availability

The authors claim that this manuscript describes an original research work that has not been preregistered. The data presented in this study are available upon request from the corresponding authors. The data are not publicly available due to compliance with privacy laws.

## Supplementary Materials

The following supporting information can be downloaded at: https://www.mdpi.com/article/10.3390/bs13050377/s1; Table S1: STROBE Statement—Checklist of items that should be included in reports of cross-sectional studies.
